# Job Demands and Resources, Burnout, and Psychological Distress of Employees in the Chinese Non-profit Sector

**DOI:** 10.3389/fpsyt.2021.790064

**Published:** 2021-12-15

**Authors:** Guosheng Deng, Chienchung Huang, Shannon P. Cheung, Congcong Zhang

**Affiliations:** ^1^School of Public Policy and Management, Tsinghua University, Beijing, China; ^2^School of Social Work, Rutgers, The State University of New Jersey, New Brunswick, NJ, United States; ^3^Department of Youth Work Research, China Youth University of Political Studies, Beijing, China

**Keywords:** job demands, job resources, burnout, psychological distress, non-profit

## Abstract

The non-profit sector in China has expanded significantly in the past few decades. However, employees in non-profits experience high burnout rates, indicating a need to study non-profit work conditions and their effect on employees. This study applies the job demands and resources (JD-R) model and examines the effects of job demands (JD) and job resources (JR) on burnout and psychological distress experienced by non-profit employees, recruited via quota sampling, across China (*n* = 233). The findings from path analysis showed that JR had strong and negative effects on burnout and on psychological distress, while JD had strong and positive effects on burnout and on psychological distress. Burnout partially mediated the relations between JD-R and psychological distress. These results highlight the importance of JD-R in reducing burnout and psychological distress in non-profit employees in China. Research and practice implications are discussed.

## Introduction

Around the globe, individuals employed in human services experience significant burnout (1–3). This raises concern for the sustainability of these important professions, given that a multitude of cross-cultural studies have shown a positive association between burnout and turnover (4–6). High turnover in human services can lead to several negative outcomes for workers and clients alike. With a dwindling labor force, human service workers may be forced to take on greater workloads, which can further exacerbate burnout and turnover (7). Moreover, individuals who experience burnout are more likely to report high psychological distress (8), which can affect their interactions with clients and vulnerable community members. Despite the rapid growth of China's non-profit sector (9, 10), studies have shown that non-profit employees in China experience high burnout and turnover (11, 12), potentially due to mounting job demands (13). This has in turn led to rising recruitment pressure and organizational instability, along with decreasing service quality (11, 12), prompting a dire need for research on the work conditions and outcomes of non-profit employees. With better understanding of their employees' experiences, non-profit organizations can design interventions and solutions to mitigate turnover and retain their labor force. Thus, this study applies the job demands and resources (JD-R) model (14–16) to examine burnout and psychological distress in a sample of Chinese non-profit employees.

## Development of the Non-Profit Sector in China

In 2004, a shift in Chinese national policy allowed individuals to begin establishing foundations (17). With the promulgation of the Regulations on Foundation Management, foundations in China rapidly increased. In 2018, there were about 7,200 foundations in China, which was 7.4 times greater than the number of foundations that existed in 2005 (17, 18). While the unprecedented growth of foundations indicates the expanding role of philanthropy organizations in Chinese society, high rates of burnout and turnover (11, 12) may threaten the growth and sustainability of the philanthropy sector in years to come. In 2017, over one-quarter (27.6%) of philanthropy workers in non-profit organizations left their jobs. Similarly, Tsinghua University Philanthropy Research Institute (11) found that just over one-fifth (20.2%) of employees in non-profit organizations have the intention to resign (11).

## The JD-R Model, Burnout, and Psychological Distress

The JD-R model posits that the work conditions can be divided into job demands (JD) and job resources (JR), and workers' health and outcomes are differentially affected by each (15, 16). The former describes aspects of the job that require a sustained physical or mental effort from an individual. The required effort to meet these JD is thought to come with a physiological or psychological cost, leading to a state of exhaustion or fatigue. However, the presence of JR, job aspects that can help facilitate achievement of work goals, are thought to mitigate the different costs of JD. Through the energy-driven process and the motivation-driven process, JD-R can lead to or protect against employee burnout (14, 15).

Burnout is often positively associated with negative work outcomes such as turnover intentions and low job satisfaction (19–21). Burnout is generally recognized as an occupational hazard specific to human service professionals, whose occupations are characterized by chronically taxing emotional demands (15, 22, 23). Burnout can also lead to poorer individual well-being and functioning, including psychological distress, an emotional state of deep discomfort characterized by symptoms of depression and anxiety (24–26). Psychological distress in human services professionals has serious implications for employee health and agency outcomes. It can positively predict serious mental illness and alcohol and substance use, as well as work outcomes like absenteeism, turnover, and anxiety disorders (27–29).

Cross-discipline (6, 14, 16) and cross-cultural studies (23, 30, 31) applying the JD-R model have found strong associations between JD-R and work outcomes, including burnout, stress, work engagement, and health (32–35). These findings have been consistent with the dual process theorized by Bakker et al. (14). JD acts as significant stressors that upend individuals' health and functioning that lead to psychological distress (24–26). In fact, JD-R and psychological distress have been studied together cross-culturally (36–38). These studies' results found a strong positive correlation between JD and psychological distress. For example, Ben-Ezra and Hamama-Raz found that work demands significantly and directly affected psychological distress (*r* = 0.23, *p* < 0.001) among over 600 social workers during the COVID-19 pandemic (36).

While psychological distress has been studied among samples in various occupations (39–42), to the authors' knowledge, no studies have investigated psychological distress among non-profit employees specifically, despite the growth of non-profit sectors around the globe [e.g., (43, 44)] and especially in China (17, 18). Thus, we seek to fill this gap in the literature by examining psychological distress among Chinese non-profit employees, including its relations with JD-R and their underlying mechanism. The results of this study can support the development of interventions that aim to reduce burnout and psychological distress of employees in the evolving non-profit sector in China, thereby aiding in the retainment of this important labor force.

## Hypotheses

**Figure 1:**
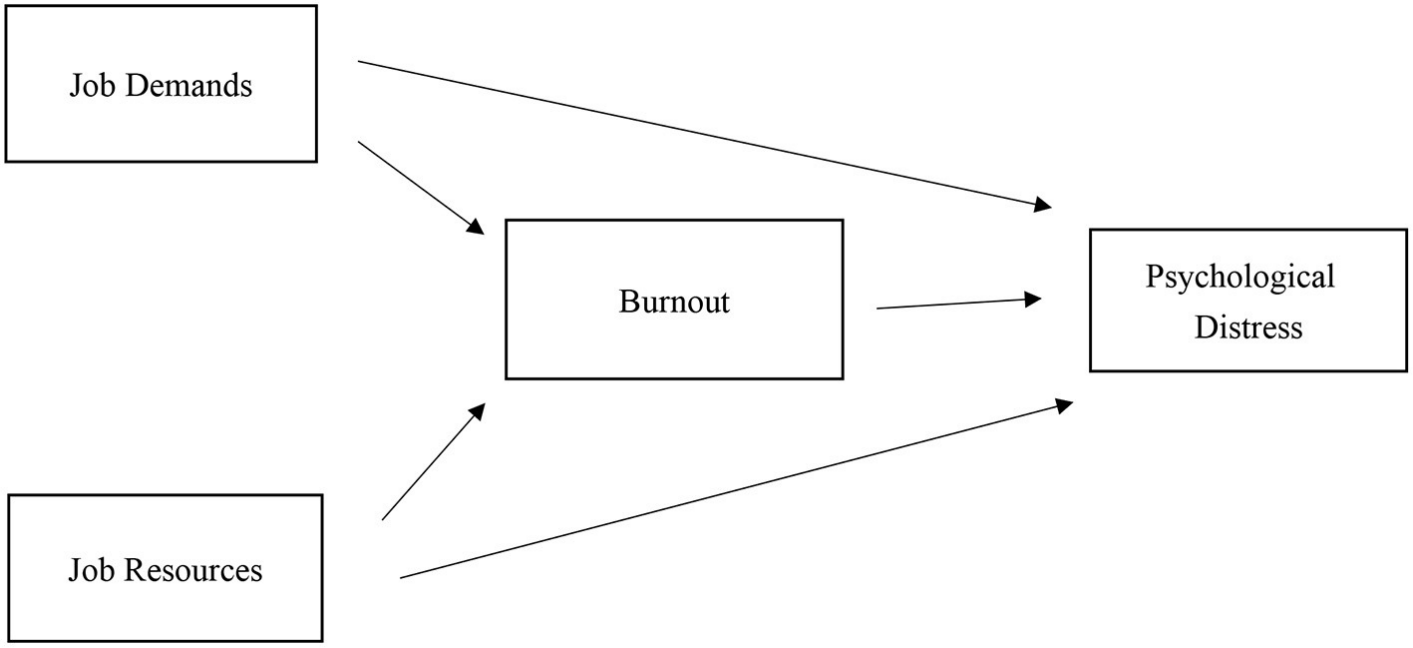
Conceptual model of JD-R, burnout, and psychological distress.

Based on the JD-R model, we hypothesized a mediational pathway by which JD-R affects psychological distress via burnout, as shown in Figure 1. Our hypotheses are as follows: (1) JD-R will affect burnout differentially, with JD having a positive association with burnout and JR having a negative association with burnout. (2) JD-R will also have differential effects on psychological distress. JD and psychological distress will be positively associated, while JR and psychological distress will be negatively associated. (3) JD-R will indirectly affect psychological distress via burnout, indicating burnout's mediational effect on the relation between psychological distress and burnout.

## Methods

### Data and Sample

Data were collected from foundation employees via an anonymous web-based survey that was hosted on the WJX platform (https://www.wjx.cn/), one of the largest and most reliable web-based survey platforms in China. The survey was administered by a research center at Tsinghua University's School of Public Administration, the Social Innovation and Rural Revitalization Research Center (SIRRRC). SIRRRC had collaborated with Guangdong Guoqiang Foundation and China Foundation Development Forum to provide trainings for employees that came from 270 local foundations in March 2021. SIRRRC used quota sampling for the survey. Given that 95.8% of foundations in China are local foundations while 4.2% are national foundations (11), SIRRRC randomly selected 12 additional national foundations to sample with the 270 local foundations that were receiving training at SIRRRC, resulting in a final sample of 282. SIRRRC sent out invitations, with a link to the survey hosted on WJX, to secretary-generals of the 282 foundations on May 20, 2021, and asked them to have their employees participate in the online survey. SIRRRC sent reminders to participate in the survey 7 and 14 days after the initial invitation was sent to the foundations. The survey was closed on June 20, 2021, and SIRRRC had received 233 responses by then. An informed consent process was implemented prior to the survey. Following participation in the survey, respondents randomly drew a red envelope which contained between 0 and 18 RMB (5 RMB on average, or 1 USD). The research protocol was approved by the research review committee by the Huamin Research Center at Rutgers University (HRCProtocol #2021002) and by SIRRRC on April 21, 2021.

### Measures

The dependent variable, psychological distress, was assessed via the K6 (45, 46), which consists of six questions that ask respondents about past 30-day prevalence of emotions that indicate psychological distress: nervousness, hopelessness, restlessness, depression, and worthlessness. Participants identified how frequently they felt each of these emotions on a 4-point Likert scale. Zero indicated “none of the time,” while four indicated “all of the time.” An additional item asked respondents the frequency at which they perceived that “everything was an effort.” Psychological distress was calculated by taking the sum of all item responses. Thus, total psychological distress scores had a possible range of 0–24. As in past studies [e.g., (29, 38, 47)], the K6 showed high reliability in our sample of foundation employees. The Cronbach's alpha of the 6 items was 0.91.

Burnout was assessed by the Oldenburg Burnout Inventory [OLBI; (48)], a 16-item scale divided into two subscales, exhaustion and disengagement from work. Each subscale contains 8 items, which are worded in the positive direction (4 items) and negative direction (4 items). Exhaustion encompasses both cognitive and physical fatigue. Disengagement describes the phenomenon of an individual experiencing distance from their work, work object, and work content. Responses followed a 4-point Likert scale, and participants were instructed to indicate the degree to which they strongly agreed (4) or strongly disagreed (1) with each item. Responses to the 8 positively worded items (4 in each subscale) were reversed to ensure that higher scores represented greater burnout. Burnout was calculated by taking the average of all item responses. In this study, the Cronbach's alpha of OLBI was 0.83.

We adapted subscales from Questionnaire sur les Ressources et Contraintes Professionnelles [QRCP; (31)] to measure JD-R in our sample. Based on the nature of non-profit work, we prioritized the adaptation of three JD dimensions and three JR dimensions. The JD dimensions measured in this study were as follows: workload, emotional workload, and changes in tasks. For JR, we measured relationship with colleagues, relationship with supervisor, and information on job performance. In the QRCP (31), each dimension is measured via 4 items, and item responses follow a 7-point Likert scale that ranges from 1 (never) to 7 (always). Higher scores indicate that the respondent experiences greater JD or has more JR at their disposal. JD and JR scores were calculated by taking the average of item responses. Lequeurre et al. (31) reported high reliability for each dimension, with all yielding a Cronbach's alpha value over 0.80. In this study, the Cronbach's alphas were 0.82 and 0.93 for JD and JR, respectively.

### Analytical Approach

Analysis began with descriptive analyses to observe sample characteristics. We also conducted correlation analyses to examine the relations among JD-R, burnout, and psychological distress. Then, in order to test whether burnout had any mediating effect on the relations between JD-R and psychological stress, we conducted path analysis. Path analysis was selected over regression analysis because it allows for simultaneous examination of direct and indirect effects through mediating variables. All analyses were conducted using STATA software 16.0. Finally, we conducted regression analyses of models that included covariates such as individual demographic information and organizational characteristics. The estimates of regression analyses were similar to those of our path analysis. These results are not displayed in the current paper but are available from the corresponding author upon request.

## Results

We display the results of descriptive analyses in Table 1. The study sample had moderate burnout (*M* = 2.3; *SD* = 0.4) and psychological distress (*M* = 5.4; *SD* = 4.5). The sample reported relatively high JD (*M* = 4.6; *SD* = 0.8) and JR (*M* = 5.2; *SD* = 0.9). Pearson's correlation analysis indicated a positive correlation between JD and burnout as well as between JD and psychological distress. By contrast, JR had a negative correlation with burnout and psychological distress. Burnout was significantly correlated with psychological distress. Finally, JD and JR had no significant correlation with each other.

**Table 1:** Descriptive statistics and correlations of key variables.

	**Mean (S.D.)**	**1**	**2**	**3**	**4**
1. Psychological stress [0–24]	5.4 (4.5)	—			
2. Burnout [1-4]	2.3 (0.4)	0.55^***^	—		
3. Job demands [1-7]	4.6 (0.8)	0.27^***^	0.39^***^	—	
4. Job resources [1-7]	5.2 (0.9)	−0.52^***^	−0.43^***^	−0.03	—

*N = 233. Numbers in brackets show ranges of the variables*.

*** *p < 0.001*.

**Figure 2:**
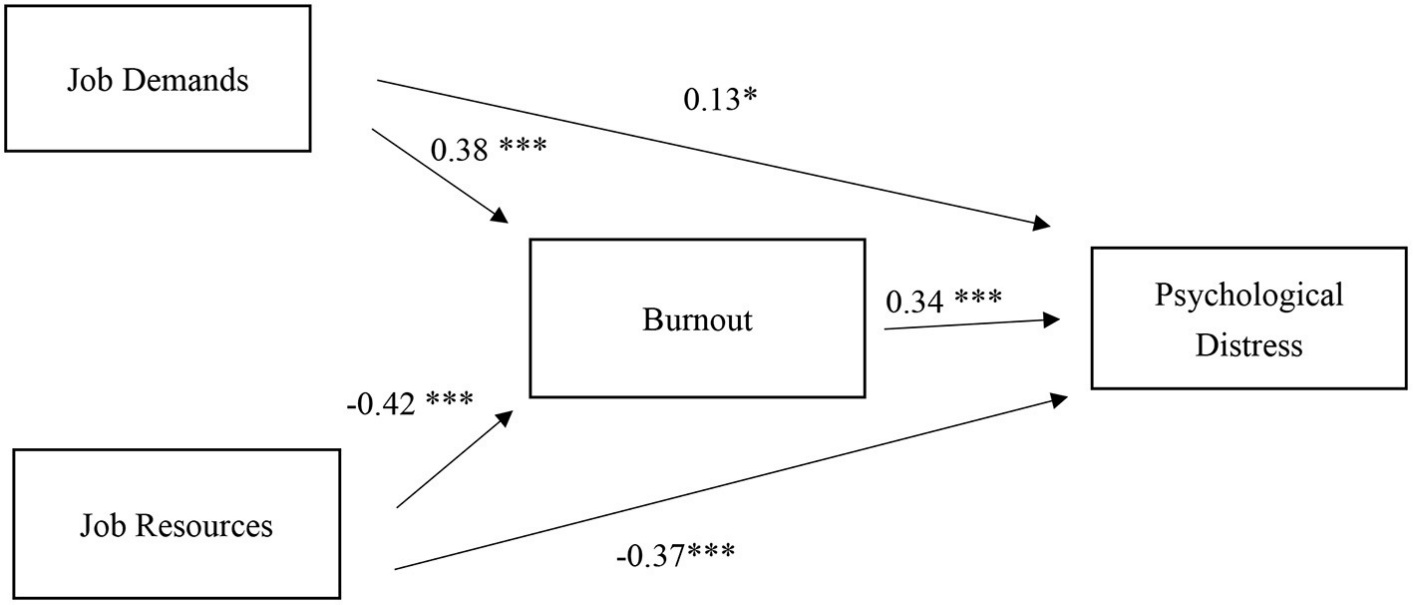
Standardized estimates of JD-R, burnout, and psychological distress model. *p < 0.05, ***p < 0.001.

In Figure 2, we present the standardized estimates from path analysis. JD-R had significant associations with burnout. JR was negatively associated with burnout (β = −0.42, *p* < 0.001), while JD was positively associated with burnout (β = 0.38, *p* < 0.001). These findings support hypothesis 1. Both JD (β = 0.13, *p* < 0.05) and burnout (β = 0.34, *p* < 0.001) had significant and positive associations with psychological distress. JR, on the other hand, had a significant and negative association with psychological distress (β = −0.37, *p* < 0.001). These findings confirm hypothesis 2.

JD had a total effect of 0.26 (*p* < 0.001) on psychological distress. JD had an indirect effect of 0.13 on psychological distress through burnout (*p* < 0.001). Burnout mediated 0.50 (0.13/0.26) of JD's effect on psychological distress. JR, on the other hand, had a total effect of −0.51 on psychological distress (*p* < 0.001) and an indirect effect of −0.14 on psychological distress through burnout (*p* < 0.001). This indirect effect accounted for 0.27 of the total effect (−0.14/−0.51). These findings are consistent with our hypotheses, suggesting that burnout partially mediated the associations between JD-R and psychological distress. These findings are consistent with hypothesis 3.

## Discussion

The descriptive findings indicate that Chinese non-profit employees faced high JD but also had many JR available to them, compared to previous study (31). Estimates from path analysis were consistent with our hypotheses, which applied previous findings on JD-R's dual process and posited that JD-R would differentially affect burnout in our sample of non-profit employees from China (hypothesis 1). The first process, the health-impairment or energy depletion process, was indicated by the significant positive association between JD and burnout. Meanwhile, the second process, the motivation process, was indicated by the negative association between JR and burnout. Thus, non-profit employees who can utilize their organization's JR may mitigate their risk of experiencing burnout. The magnitude of the estimates produced by our analyses suggests that JR have a greater effect on burnout in Chinese non-profit employees than do JD. Based on this, it is likely that the lack of JR, such as positive relationships with colleagues and/or supervisors or availability of job-related information, can be highly detrimental to the health of non-profit employees in China, more so than JD like workload, emotional workload, and changes in tasks.

The findings are consistent with previous studies in this area. Compared to employees in the private and public sectors, non-profit employees tend to have low extrinsic rewards (e.g., fair pay and financial well-being) and high JR (e.g., helpful coworkers and supervisors) (49–52). Studies found that non-profit employees were more sensitive to JR than employees in public and private sectors (50, 52, 53). For example, Stater and Stater used 2002–2014 data from the General Social Survey (GSS) and found that having helpful coworkers and supervisors both had larger effects on job satisfaction for non-profit workers than for private sector and public sector workers. Helpful supervisors were related to less turnover intent to a greater extent in the non-profit sector than in the for-profit and public sectors (52).

In the current study, our sample of 233 non-profit employees reported having relatively high JR available to them, but these numbers must be understood within the context of the growing non-profit sector of China. Resource availability generally varies by foundation type (9, 11, 18). A small local foundation, for example, may be limited in the JR that they can provide to their employees. By contrast, national foundations, with more access to resources, may be better equipped to provide supportive JR to their employees. Given this limitation, future research may examine other antecedents of burnout and psychological distress in non-profit employees so as to investigate other interventions points that may inspire less costly ways of supporting the well-being of non-profit employees, particularly those working in smaller local foundations.

Still, increasing JR does not necessarily mean that non-profit agencies may simultaneously continue to project significant JD on their employees. Employers must be wary of their employees' JD. After all, path analysis showed that both JD and JR important predictors of psychological distress in non-profit employees (hypothesis 2). These relations were in line with previous findings from other studies' application of the JD-R model to samples of professionals from other occupational groups, showing that JD-R are important predictors of burnout and health outcomes (14, 16, 33). Our study results extend past literature by providing support for the application of JD-R in studying burnout and subsequent psychological distress in non-profit employees in China, a relatively understudied but fast-growing occupational group.

The significant indirect effects of JD-R on psychological distress via burnout (hypothesis 3) and the strong positive direct effect of burnout on psychological distress suggest that reducing burnout can also reduce psychological distress and its associated risks, such as mood and anxiety disorders (27, 29). Our results therefore underscore a need to buffer the effects of JD on burnout and psychological distress. Rather than relying on resource-constrained non-profit agencies to increase JR, less costly interventions may be considered. Scholars have hinted at the cost-effectiveness of administering stress-reducing worksite interventions, such as mindfulness-based interventions (MBI's) (54, 55). Bartlett et al.'s (54) systematic review and meta-analysis found that there is certainly potential in the use of workplace mindfulness trainings to reduce psychological distress and promote well-being of employees. Many studies have emphasized the effectiveness of mindfulness-based stress reduction (MBSR), mindfulness-based cognitive therapy (MBCT), and MBI's to reduce psychological distress and increase well-being (56–58), and MBSR and MBCT have both shown promise as workplace interventions for occupational groups like physicians, teachers, and psychotherapists [see (59) for review]. In consideration of the resource constraints of local foundations in China, it will be necessary to garner empirical evidence in support of the cost-effectiveness and efficacy of MBI's to promote non-profit employee well-being, which, ultimately, may mitigate burnout, psychological distress, and turnover.

Lastly, the results of this study must be taken with the context of a few limitations. Since we collected and analyzed cross-sectional data, our results may approximate associative relations among JD-R, burnout, and psychological distress rather than causal relations. This limitation may be addressed by the collection and analysis of longitudinal data. Second, unobserved variables, such as personal experience and traits, could also affect JD-R, burnout, and psychological distress. For example, studies have shown that burnout might be related to post-traumatic stress symptoms, temperament, alexithymia, and resilience (60, 61). Because these were not included in our model, they may have unknown effects on the estimates from our analysis. Another limitation of this study is that all data on our key variables were reliant on foundation employees' self-reports and are therefore subject to reporting errors. Future studies may address this limitation through the use of data triangulation, perhaps gathering additional data from multiple employees from the same foundations as well as from people with a range of job positions and employees' family members. Finally, the sample in this study was drawn mostly from individuals who were largely participants of an online training hosted by Tsinghua University in 2021. As such, these employees may share similar unobserved characteristics that may affect the estimates produced by our analysis, and the generalizability of our results to all foundation employees in China is unknown. A future study may seek to study a more representative sample to better draw conclusions about JD-R, burnout, and psychological distress in all non-profit sector employees in China.

## Conclusion

The findings in this study indicate that JD significantly increase burnout, and JR significantly decrease burnout in a sample of non-profit employees in China. Importantly, JR appeared to have larger effects on burnout than did JD, indicating a need to focus on improving the accessibility of JR to non-profit employees in China. We further expand the literature on JD-R and health outcomes by providing empirical evidence of the mediational effects that burnout has on the relations between JD-R and psychological distress. In doing so, we provide support for interventions that protect against the deleterious effects of JD while also promoting JR's protective effects on non-profit employees' health.

## Data Availability Statement

The raw data supporting the conclusions of this article will be made available by the authors, without undue reservation.

## Ethics Statement

The studies involving human participants were reviewed and approved by Research Review Committee, Huamin Research Center at Rutgers University. Written informed consent for participation was not required for this study in accordance with the national legislation and the institutional requirements.

## Author Contributions

GD and CH: conceptualization, resources, investigation, and data curation. GD, CH, SC, and CZ: methodology, software, validation, formal analysis, and writing—original draft preparation. All authors contributed to the article and approved the submitted version.

## Conflict of Interest

The authors declare that the research was conducted in the absence of any commercial or financial relationships that could be construed as a potential conflict of interest.

## Publisher's Note

All claims expressed in this article are solely those of the authors and do not necessarily represent those of their affiliated organizations, or those of the publisher, the editors and the reviewers. Any product that may be evaluated in this article, or claim that may be made by its manufacturer, is not guaranteed or endorsed by the publisher.
